# Psychometric Validation of the Kazakh Version of the Patient Health Questionnaire-9 (PHQ-9)

**DOI:** 10.3390/healthcare14172713

**Published:** 2026-08-25

**Authors:** Nursultan Seksenbayev, Timur Moldagaliyev, Lazzat Zhamaliyeva, Akmaral Baspakova, Assel Baibussinova, Nabi Yessimov, Kamila Akkuzinova, Yelaman Toleuov, Shynargul Zhakiyeva, Sandugash Kudaibergenova, Ken Inoue

**Affiliations:** 1 Department of Psychiatry and Narcology, Semey Medical University, Semey 071400, Kazakhstan; nurs_7sk@inbox.ru (N.S.); timur_party@inbox.ru (T.M.); akkuzinova@gmail.com (K.A.); elamantol96@gmail.com (Y.T.); 2 Department of General Practice No. 2, West Kazakhstan Marat Ospanov Medical University, Aktobe 30012, Kazakhstan; lzhamalieva@mail.ru; 3 Department of Epidemiology, West Kazakhstan Marat Ospanov Medical University, Aktobe 30012, Kazakhstan; baspakova@zkgmu.kz; 4 Department of Epidemiology and Biostatistics, Semey Medical University, Semey 071400, Kazakhstan; assel.baibussinova@smu.edu.kz; 5 Caspian International School of Medicine, Caspian University, Almaty 050040, Kazakhstan; nabi_es@mail.ru; 6 Department of General Practice No. 1, West Kazakhstan Marat Ospanov Medical University, Aktobe 30012, Kazakhstan; zhakiyevas@inbox.ru; 7 Department of General and Applied Psychology, Al-Farabi Kazakh National University, Almaty 050040, Kazakhstan; sandugash.kudaibergenova@kaznu.kz; 8 Health Service Center, Kochi University, Kochi 780-8520, Japan

**Keywords:** depressive symptoms assessment, PHQ-9, psychometric validation, cross-cultural adaptation, Kazakh language, confirmatory factor analysis, mental health assessment, primary care

## Abstract

Background: Depression is a major global health burden, requiring culturally appropriate assessment instruments, but no validated Kazakh-language version of the Patient Health Questionnaire-9 (PHQ-9) is available. This study aimed to translate, culturally adapt, and evaluate the psychometric properties of the Kazakh PHQ-9 in a community-based, non-clinical sample. Methods: This cross-sectional study included 503 Kazakh-speaking adults (mean age = 24.0 years, SD = 10.1; 51.3% women and 48.7% men) who completed the Kazakh PHQ-9 and Generalized Anxiety Disorder-7 (GAD-7) questionnaires. Internal consistency, structural validity, measurement invariance across genders, and convergent validity were assessed. Results: Internal consistency was excellent (Cronbach’s α = 0.91), and both one-factor and correlated two-factor models showed excellent fit (CFI = 0.998; TLI = 0.997–0.998; RMSEA = 0.046–0.048; SRMR = 0.050–0.052), with a slightly better fit for the latter. Measurement invariance across genders was supported, and PHQ-9 and GAD-7 scores were strongly correlated (r = 0.68, *p* < 0.001). Conclusions: The Kazakh PHQ-9 demonstrated excellent internal consistency and structural validity, measurement invariance across genders, and strong convergent validity in this non-clinical sample. These findings support its use for assessing depressive symptoms in Kazakh-speaking adults. Clinical validation is needed to establish diagnostic validity and screening performance.

## 1. Introduction

Depression is among the leading contributors to disability worldwide and represents a major public health concern [1,2]. Reliable and culturally appropriate instruments are important for the early identification and assessment of depressive symptoms across diverse populations. The Patient Health Questionnaire-9 (PHQ-9) is a brief self-report instrument developed to assess depressive symptoms based on the diagnostic criteria for major depressive disorder [3]. Owing to its brevity and ease of administration, it has become one of the most widely used depression screening instruments in clinical practice and epidemiological research [4,5,6].

Numerous validation studies conducted across different countries and populations have supported the reliability and validity of the PHQ-9 in both community and clinical settings [7,8,9,10,11]. However, findings regarding its factorial structure have not been consistent, with some studies supporting a unidimensional model and others distinguishing cognitive–affective and somatic symptom domains [12,13]. Methodological research has also indicated that fit indices such as the RMSEA may produce inflated values on short scales with limited degrees of freedom, even when other goodness-of-fit indices suggest an acceptable model fit [14]. These methodological challenges may be particularly relevant in non-clinical community samples because positively skewed symptom distributions, floor effects, and low endorsement of severe symptoms may affect the evaluation of factorial models.

Despite the extensive international validation of the PHQ-9, direct translation of psychometric instruments without systematic cultural adaptation may compromise their validity and interpretability. Measurement bias may also arise when linguistic nuances or culturally specific expressions of distress are not adequately captured [15]. The COSMIN framework is internationally recognized as a reference standard for evaluating the measurement properties of health-related instruments and provides a rigorous methodological basis for cross-cultural adaptation and psychometric validation [16]. Such adaptation is particularly important for self-report mental health instruments because linguistic and sociocultural differences may influence symptom interpretation, response patterns, and factorial structure [15,16].

Despite the increasing use of the Kazakh language in healthcare and public health services, validated Kazakh-language mental health assessment instruments remain limited. In particular, no culturally adapted and psychometrically validated Kazakh version of the PHQ-9 was previously available, restricting standardized assessment of depressive symptoms in Kazakhstan. The availability of such an instrument may support culturally appropriate assessment and facilitate comparability between studies conducted in Kazakhstan and international psychometric research.

Therefore, the present study aimed to translate and culturally adapt the PHQ-9 into Kazakh and to evaluate its preliminary psychometric properties in a community-based, non-clinical sample of Kazakh-speaking adults. Specifically, we examined internal consistency, structural validity, measurement invariance across genders, and convergent validity with the GAD-7. We hypothesized that the Kazakh version of the PHQ-9 would demonstrate adequate internal consistency and structural validity, measurement invariance across genders, and a moderate-to-strong positive correlation with the GAD-7 (r ≥ 0.50), supporting convergent validity.

## 2. Materials and Methods

According to official information provided by the PHQ Screeners platform and original publications, the PHQ-9 is freely available for non-commercial academic use, and no formal permission is required to reproduce, translate, culturally adapt, or publish this instrument, provided that appropriate attribution to the original authors is given. The original authors were fully acknowledged in accordance with recommended citation practices [Spitzer RL, Kroenke K, Williams JBW. Patient Health Questionnaire (PHQ) Screeners]. Available at https://www.phqscreeners.com (last accessed 13 April 2026).

### 2.1. Study Design

This study used a community-based cross-sectional design to translate, culturally adapt, and evaluate the psychometric properties of the Kazakh version of the PHQ-9. Data were collected at a single time point from Kazakh-speaking adults using self-administered questionnaires. Content validity was not inferred from the cross-cultural adaptation process alone; instead, preliminary support for content validity was obtained through expert review and cognitive debriefing with representatives of the target population, focusing primarily on item relevance, conceptual equivalence, and comprehensibility. The psychometric evaluation included internal consistency, structural validity, measurement invariance across genders, and convergent validity with the GAD-7. The study did not include diagnostic validation procedures, such as structured psychiatric interviews or analyses of diagnostic accuracy.

### 2.2. Participants

A total of 503 participants were recruited via convenience sampling from the general community. The adequacy of the achieved sample size was evaluated according to COSMIN recommendations for factor analysis, which indicate that a very good sample size should include at least seven participants per item and no fewer than 100 participants overall. For the nine-item PHQ-9, the applicable minimum was therefore 100 participants. The final sample of 503 participants, corresponding to approximately 56 participants per item, substantially exceeded this criterion. Both gender groups also included more than 100 participants, supporting the adequacy of the sample for the planned confirmatory factor and measurement invariance analyses.

The eligibility criteria were age ≥ 18 years, sufficient ability to read and understand the Kazakh-language study information and questionnaires, and provision of electronic informed consent. Respondents were not included in the analytical dataset if they did not meet the age or language eligibility criteria, did not provide informed consent, or did not submit a complete questionnaire. The online survey required responses to all questionnaire items before submission; therefore, the final analytical dataset contained no incomplete records. No formal cognitive screening was conducted, and cognitive impairment was therefore not specified as a formal exclusion criterion.

Participants accessed the survey through online distribution channels, including social media platforms and messaging applications. Therefore, the sample cannot be considered representative of the broader Kazakh population. Individuals aged ≥ 18 years were eligible, and the mean age of the participants was 24.0 years (SD  =  10.1). The gender distribution was balanced, with 258 women (51.3%) and 245 men (48.7%).

Participation was voluntary and anonymous, and all respondents provided informed consent prior to completing the study’s questionnaires. Since the study sample consisted of individuals from the general population, we were able to assess the psychometric properties of the Kazakh version of the PHQ-9 in a non-clinical context.

### 2.3. Instruments

The PHQ-9 is a self-report measure designed to assess the severity of depressive symptoms and was originally developed based on the diagnostic criteria for major depressive disorder.

The scale consists of nine items used to assess the frequency of depressive symptoms experienced over the past 2 weeks. Each item is rated on a four-point Likert scale ranging from 0 (“not at all”) to 3 (“nearly every day”), yielding a total score of 0 to 27, with higher scores indicating greater severity of depressive symptoms.

Previous studies have reported acceptable psychometric properties of the PHQ-9 across diverse cultural contexts and community and clinical populations. In the present study, the Kazakh version of the PHQ-9 was used following a standardized translation and cross-cultural adaptation procedure.

The Generalized Anxiety Disorder-7 (GAD-7) is a seven-item self-report instrument designed to assess anxiety symptom severity over the preceding two weeks. Each item is rated from 0 (“not at all”) to 3 (“nearly every day”), yielding a total score ranging from 0 to 21, with higher scores indicating greater anxiety symptom severity. Scores of 5, 10, and 15 represent mild, moderate, and severe symptom levels, respectively. In the original validation study, the GAD-7 demonstrated excellent internal consistency (Cronbach’s α = 0.92), good test–retest reliability (intraclass correlation coefficient = 0.83), and evidence of criterion, construct, and factorial validity [17]. In the present study, the GAD-7 total score was used specifically to assess the convergent validity of the Kazakh version of the PHQ-9.

### 2.4. Translation and Cultural Adaptation

Two independent forward translations of the PHQ-9 from English into Kazakh were performed by a clinician with expertise in mental health and a professional linguist, working independently. The translations were compared and synthesized into a single reconciled version through a consensus process, prioritizing conceptual equivalence, linguistic clarity, and cultural appropriateness, with particular attention to sensitive items.

The reconciled version was independently back-translated into English by two translators blinded to the original PHQ-9 and not involved in the forward translation stage. The back-translations were compared with the source instrument to evaluate semantic and conceptual correspondence. No meaningful discrepancies were identified, and the reconciled Kazakh version was retained without modification.

The pre-final Kazakh version of the PHQ-9 was reviewed by an expert committee consisting of mental health clinicians, a linguist, and healthcare professionals familiar with the target population. The committee evaluated the comprehensibility, cultural relevance, and conceptual equivalence of each item. Minor wording refinements were discussed where necessary, and consensus was reached on the final version. The expert review provided preliminary evidence regarding item relevance, cultural appropriateness, comprehensibility, and conceptual equivalence; however, it was not considered sufficient to confirm content validity in isolation.

The translation and cross-cultural adaptation process followed the COSMIN (COnsensus-based Standards for the selection of health Measurement INstruments) recommendations for patient-reported outcome measures. Detailed documentation of the forward translation, reconciliation, and back-translation stages is provided in the Supplementary Materials (Tables S1–S3).

Cognitive debriefing was conducted with 34 representatives of the target population to evaluate the comprehensibility and cultural appropriateness of the Kazakh version of the PHQ-9. Participants were asked to paraphrase each item and comment on the clarity and interpretation of the items, response options, and instructions. As reported in Supplementary Table S4, all PHQ-9 items, response options, and instructions were generally well understood, and no participants reported comprehension difficulties. Consequently, no substantive modifications were required, and the pre-final version was retained as the final Kazakh version. These findings provided direct evidence of comprehensibility. However, comprehensiveness was not formally evaluated as a separate component; therefore, the expert review and cognitive debriefing were interpreted as providing preliminary support rather than definitive confirmation of content validity.

The translated version was subsequently evaluated within a psychometric validation framework that is consistent with the approaches used in studies examining the measurement properties of the PHQ-9 in clinical and research settings [6].

The final Kazakh version of the PHQ-9 questionnaire is provided in the Supplementary Materials (File S1).

### 2.5. Data Collection

Participants’ data were collected via an online survey administered through a web-based questionnaire platform. Participants were recruited through convenience sampling by distributing the survey link via social media platforms and messaging applications to reach the general population. Data were collected from February 12 through March 16, 2026. A total of 503 consenting participants submitted complete questionnaires, all of which were included in the final analysis. Because the survey was distributed through an open anonymous link and the online survey platform did not record unique survey accesses or unsubmitted questionnaires, the denominator required to calculate the response rate was unavailable.

Potential participants accessed the questionnaire through an anonymous link and provided informed consent before completing the survey. The questionnaire included demographic questions, followed by the Kazakh version of the PHQ-9. All responses were recorded electronically and stored in a secure database for subsequent statistical analysis. No missing data were present in the final analytical dataset because participants were required to complete all questionnaire items before submitting the online survey. This approach facilitated recruitment across different regions of Kazakhstan; however, the use of convenience-based online sampling may have led to self-selection bias and overrepresentation of younger individuals and participants with greater digital access and higher educational attainment. Therefore, the findings should be interpreted with caution regarding generalizability to the broader Kazakh population.

### 2.6. Ethical Considerations

This study was approved by the Bioethics Committee of the West Kazakhstan Marat Ospanov Medical University (Protocol 4, Registration 04.01/01, Meeting Date: 26 April 2024). All participants provided informed consent prior to participation.

The survey was anonymous and did not collect personally identifiable information. Although the PHQ-9 includes an item related to thoughts of self-harm, participants were informed that the questionnaire was intended for research purposes and that individuals experiencing psychological distress should seek professional support from mental health services.

### 2.7. Statistical Analyses

All statistical analyses were performed using R statistical software (version 4.6.1; R Foundation for Statistical Computing, Vienna, Austria). Descriptive statistics were calculated for demographic characteristics and PHQ-9 item responses. Continuous variables are summarized using means and standard deviations (SDs), whereas categorical variables are summarized using frequencies and percentages. The total PHQ-9 score was additionally characterized using the median, interquartile range, and observed score range. The distributions of the individual PHQ-9 item responses were evaluated using skewness and kurtosis.

Internal consistency reliability was assessed using Cronbach’s alpha, ordinal McDonald’s omega (ω), and corrected item–total correlations. Given the ordinal nature of the PHQ-9 items, McDonald’s omega was estimated from a polychoric correlation matrix.

Because the PHQ-9 consists of ordered categorical items with four response categories, confirmatory factor analysis (CFA) was conducted using the weighted least squares mean and variance adjusted (WLSMV) estimator, which is currently recommended over maximum likelihood estimation for structural equation modeling with ordered categorical indicators [18,19]. Accordingly, WLSMV was selected as the primary estimation method because it provides more appropriate parameter estimates and model fit statistics for ordinal response data. CFA was performed using the lavaan package (version 0.6-21) implemented through the lavaangui interface (version 0.4.0) [20]. Two competing measurement models were evaluated based on previous psychometric studies: (1) a one-factor model including all nine PHQ-9 items and (2) a correlated two-factor model consisting of a cognitive–affective factor (items 1, 2, 6, 7, 8, and 9) and a somatic factor (items 3, 4, and 5).

Model fit was evaluated using the Comparative Fit Index (CFI), Tucker–Lewis Index (TLI), Root-Mean-Square Error of Approximation (RMSEA) with its 90% confidence interval (CI), and the Standardized Root-Mean-Square Residual (SRMR). Based on commonly applied methodological recommendations, CFI and TLI values ≥ 0.90 were considered indicative of acceptable fit and values ≥ 0.95 of excellent fit; RMSEA values ≤ 0.08 indicated acceptable fit and values ≤ 0.06 indicated excellent fit; and SRMR values ≤ 0.08 indicated acceptable fit [19,21]. These thresholds were treated as general guidelines rather than rigid decision rules, and the fit indices were interpreted collectively, together with model parsimony and theoretical plausibility.

Because the models were estimated using WLSMV, which is not based on full-information maximum likelihood, likelihood-based information criteria such as the Akaike Information Criterion (AIC) and Bayesian Information Criterion (BIC) were not available. A formal chi-square difference test was also not applied, because the one-factor and correlated two-factor solutions did not constitute a regular nested comparison: the one-factor solution would require fixing the correlation between the two factors at the boundary value of 1.0. Therefore, the competing models were compared by considering the fit indices collectively, together with model parsimony, theoretical interpretability, and the magnitude of the latent factor correlation.

Measurement invariance across genders was examined using multigroup CFA with the WLSMV estimator by sequentially testing configural, metric, and scalar invariance models. Given the ordinal nature of the PHQ-9 items, threshold invariance was evaluated within the scalar invariance model by constraining both factor loadings and item thresholds to equality across gender groups. Measurement invariance was evaluated using changes in the Comparative Fit Index (ΔCFI), RMSEA (ΔRMSEA), and SRMR (ΔSRMR). Following established methodological recommendations, invariance was supported when the decrease in CFI did not exceed 0.010, the increase in RMSEA did not exceed 0.015, and the increase in SRMR did not exceed 0.030 for metric invariance and 0.010 for scalar/threshold invariance [22].

Convergent validity was assessed by calculating Pearson’s correlation coefficient between the PHQ-9 and GAD-7 total scores. Based on previous psychometric evidence, a moderate-to-strong positive correlation (r ≥ 0.50) was hypothesized.

All statistical tests were two-tailed, and statistical significance was defined as *p* < 0.05.

## 3. Results

### 3.1. Sample Characteristics

All 503 participants completed the questionnaires. The sociodemographic characteristics of the study participants were as follows: the average age (mean  ±  SD) was 24.0  ±  10.1; 258 (51.3%) were women; and 245 (48.7%) were men.

### 3.2. Item Descriptive Statistics

Descriptive statistics for the individual PHQ-9 items are presented in Table 1. The mean item scores ranged from 0.112 to 0.712 points. The skewness and kurtosis values indicated a positively skewed distribution of responses, which is commonly observed in community samples when depressive symptoms are assessed. The suicidal ideation item showed stronger skewness, which is consistent with findings from other PHQ-9 validation studies in general population samples.

The mean total PHQ-9 score was 3.83 (SD = 4.89), while the median was 2.00 (interquartile range: 0.00–6.50). The observed total scores ranged from 0 to 27.

The positive skewness observed for several PHQ-9 items was consistent with the expected concentration of responses in the lower categories in a community-based, non-clinical sample. Together with the four-category ordinal response format, the non-normal distribution of item responses provided further support for the use of the WLSMV estimator in the subsequent confirmatory factor analysis.

### 3.3. Reliability Analysis

The Kazakh version of the PHQ-9 demonstrated excellent internal consistency. The Cronbach’s alpha coefficient for the total scale was 0.91, and ordinal McDonald’s omega was 0.95, with both coefficients indicating excellent internal consistency. The corrected item–total correlations ranged from 0.45 to 0.76, indicating adequate item discrimination. The lowest correlation was observed for item 9, and the remaining items demonstrated moderate-to-strong correlations with the overall scale score. Detailed reliability indices are presented in Table 2.

### 3.4. Structural Validity

Confirmatory factor analysis (CFA) was performed to examine the factorial structure of the Kazakh version of the PHQ-9 using the weighted least squares mean and variance adjusted (WLSMV) estimator. Two alternative models reported in previous psychometric studies were evaluated: a one-factor model and a correlated two-factor model. Goodness-of-fit indices for both models are presented in Table 3.

The one-factor model demonstrated an excellent overall fit to the data (χ^2^ = 58.245, df = 27, CFI = 0.998, TLI = 0.997, RMSEA = 0.048, 90% CI = 0.031–0.065, SRMR = 0.052).

The correlated two-factor model also demonstrated an excellent model fit (χ^2^ = 53.251, df = 26, CFI = 0.998, TLI = 0.998, RMSEA = 0.046, 90% CI = 0.028–0.063, SRMR = 0.050). Although some fit indices were numerically slightly better than those of the one-factor model, the differences were negligible (ΔCFI = 0.000, ΔTLI = 0.001, ΔRMSEA = 0.002, and ΔSRMR = 0.002). Standardized factor loadings ranged from 0.752 to 0.904 (Table 4), with all loadings statistically significant (*p* < 0.001). The correlation between the cognitive–affective and somatic factors was very high (r = 0.962), suggesting that the two latent dimensions were not empirically distinct. Therefore, the small differences in fit indices did not provide compelling evidence that the two-factor model was superior to the more parsimonious one-factor model.

### 3.5. Measurement Invariance Across Genders

The measurement invariance across genders was evaluated using multigroup confirmatory factor analysis with the WLSMV estimator. The configural, metric, and scalar/threshold invariance models all demonstrated good overall model fit (Table 5), with the configural model showing CFI = 0.997, TLI = 0.996, RMSEA = 0.059, and SRMR = 0.055. Constraining the factor loadings to equality across gender groups produced only minor changes relative to the configural model (ΔCFI = −0.001, ΔRMSEA = +0.008, and ΔSRMR = +0.015), all of which were within the recommended criteria and supported metric invariance. The scalar/threshold model, in which both factor loadings and item thresholds were constrained to equality, demonstrated excellent fit (CFI = 0.998, TLI = 0.998, RMSEA = 0.046, SRMR = 0.055). Relative to the metric model, CFI increased by 0.002, while RMSEA and SRMR decreased by 0.021 and 0.015, respectively, indicating improvement rather than deterioration in model fit. Collectively, the changes in CFI, RMSEA, and SRMR supported scalar/threshold invariance and indicated that the factor structure, factor loadings, and item thresholds were invariant across male and female participants.

### 3.6. Convergent Validity

Convergent validity was examined by calculating Pearson’s correlation coefficient between the PHQ-9 and GAD-7 total scores. A strong positive correlation was observed (r = 0.68, *p* < 0.001), exceeding the hypothesized threshold of r ≥ 0.50 and supporting the convergent validity hypothesis.

## 4. Discussion

### 4.1. Main Findings

This study evaluated the psychometric properties of the Kazakh version of the Patient Health Questionnaire-9 (PHQ-9) in a community sample. This version demonstrated excellent internal consistency, as indicated by both Cronbach’s alpha (α = 0.91) and ordinal McDonald’s omega (ω = 0.95). For example, a German PHQ-9 validation study reported a Cronbach’s alpha of 0.88, and studies conducted in China and other populations reported values ranging from 0.86 to 0.89 [3,12,23]. These findings suggest that the Kazakh version showed high internal consistency in the present community-based non-clinical sample, comparable to that reported in other language adaptations [24].

Although the corrected item–total correlation for item 9 (r = 0.45) was lower than those observed for the remaining items, it remained within an acceptable range. This lower coefficient may partly reflect restricted response variability resulting from the lower endorsement of the sensitive construct assessed by this item in community-based, non-clinical samples. Cultural norms and reluctance to report highly sensitive experiences may also have influenced the response pattern; however, these factors were not directly assessed in the present study and should therefore be interpreted cautiously. Importantly, cognitive debriefing indicated that item 9 was understood as intended, providing no evidence that the lower correlation resulted from translation or comprehension difficulties. Further research in clinical samples is needed to examine the performance of this item across different levels of symptom severity.

Confirmatory factor analysis using the WLSMV estimator demonstrated excellent overall fit for both the one-factor and correlated two-factor models. Although the two-factor model yielded marginally more favorable fit indices, the differences were negligible. More importantly, the very high correlation between the cognitive–affective and somatic factors (r = 0.962) indicated limited empirical separation between the two dimensions. Thus, the two-factor solution may reflect the clustering of items with related content rather than two substantively distinct latent constructs. Given the nearly equivalent model fit and the principle of parsimony, the findings favor an essentially unidimensional interpretation of the Kazakh PHQ-9 and support the use of its total score in this community-based sample. Consequently, separate interpretation of the cognitive–affective and somatic subscale scores may provide limited incremental value in the present sample and should be approached cautiously until their distinct clinical or predictive utility is demonstrated. Nevertheless, future studies in independent and clinical samples should examine bifactor or hierarchical models to determine whether specific symptom domains explain meaningful variance beyond a dominant general factor. This interpretation is consistent with previous psychometric research demonstrating closely related or competing factorial structures of the PHQ-9 across different cultural settings [13,25,26].

The results of our measurement invariance analysis demonstrated that the Kazakh version of the PHQ-9 functions equivalently across male and female participants. These findings suggest that the PHQ-9 items were interpreted in a broadly comparable manner across gender groups in the present sample, supporting the appropriateness of comparing depressive symptom scores between male and female participants. Similar findings of gender invariance have been reported in previous international validation studies of the PHQ-9, suggesting the instrument’s relative stability across demographic groups [7,8,9].

The convergent validity of the Kazakh version of the PHQ-9 was supported by a strong positive correlation with the GAD-7 (r = 0.68, *p* < 0.001), which was consistent with the expected overlap between depressive and anxiety symptom dimensions [27]. At the same time, the correlation was well below unity and corresponded to approximately 46% shared variance (r^2^ = 0.46), indicating that the two instruments were related but not redundant and captured distinguishable psychological constructs. This pattern provides additional support for the construct validity of the Kazakh PHQ-9. However, because discriminant validity was not formally evaluated, conclusions regarding the distinctiveness of the constructs should remain cautious.

An additional motivation for the present study was the limited availability of culturally adapted Kazakh-language mental health assessment instruments. The availability of a psychometrically evaluated Kazakh version of the PHQ-9 may support future community-based assessment and facilitate comparability with similar validation studies conducted in other cultural contexts [28].

However, further studies involving clinical populations and diagnostic validation procedures are necessary before conclusions regarding clinical screening performance or routine clinical implementation can be established.

The cross-cultural adaptation process represented an important component of the present study. The principal linguistic consideration was to balance semantic fidelity to the original instrument with natural and idiomatic Kazakh wording, while preserving the intended recall period and the meaning of the frequency-based response options. Differences between alternative translations were reviewed by the expert panel and resolved through consensus, with particular attention to conceptual equivalence, clarity, and cultural appropriateness. Cognitive debriefing with 34 representatives of the target population subsequently demonstrated that the items, response options, and instructions were generally well understood and culturally appropriate, and no substantive modifications to the pre-final version were required (Table S4). Thus, no culturally sensitive concepts requiring additional modification were identified during cognitive debriefing. These findings provide direct evidence of comprehensibility and preliminary support for content validity; however, comprehensiveness was not formally evaluated as a separate component.

In addition, the sample size allowed evaluation of the planned psychometric models and provided preliminary stability for the estimated parameters. Our search of the relevant literature indicates that this study provides the first psychometric validation of the PHQ-9 in the Kazakh language. The availability of a culturally adapted Kazakh version of the PHQ-9 may support future psychometric research and community-based assessment of depressive symptoms in Kazakh-speaking populations.

### 4.2. Comparison with Previous Studies

The internal consistency of the Kazakh version of the PHQ-9 observed herein (α  =  0.91) is comparable to that reported in validation studies conducted in different countries. For example, Cronbach’s α values ranging from 0.86 to 0.89 have been reported in validation studies conducted in China and the United Kingdom [3,12,13]. A comparison of psychometric properties across selected validation studies is presented in Table 6.

### 4.3. Implications for Future Research and Assessment

The availability of a culturally adapted Kazakh version of the PHQ-9 provides a foundation for future community-based assessment and psychometric research. However, further validation in clinical populations is required before the instrument can be recommended for diagnostic screening or routine clinical implementation. Future studies should evaluate test–retest reliability over an appropriate time interval, criterion validity against structured clinical interviews, diagnostic accuracy, and responsiveness to change following therapeutic interventions. Additional research should examine differential item functioning across demographic and clinical groups, including age, gender, educational level, region, and clinical status. Cross-cultural measurement invariance should also be evaluated by comparing the Kazakh version with other language versions of the PHQ-9 in multilingual or cross-national samples. These investigations would clarify whether individual items and total scores function comparably across populations and would provide a more comprehensive understanding of the instrument’s measurement properties. The use of internationally recognized instruments may ultimately facilitate comparisons between studies conducted in Kazakhstan and those performed in other cultural settings.

### 4.4. Study Limitations

Several limitations should be considered when interpreting the findings of this study. First, the study sample was recruited through convenience-based online sampling, which may limit the representativeness and generalizability of the findings. The sample consisted predominantly of younger individuals with internet access and potentially higher educational attainment, which may not fully reflect the broader Kazakh population’s demographic composition. Accordingly, the generalizability of the findings to older adults and individuals with lower digital literacy or limited internet access remains uncertain. In addition, voluntary online participation may have introduced self-selection bias.

Second, the study relied exclusively on self-report instruments administered within the same online survey. Consequently, the observed associations, including the correlation between the PHQ-9 and GAD-7, may have been influenced by common method bias. Social desirability and other response biases may also have affected participants’ reporting, particularly for sensitive symptoms. Future studies should incorporate clinician-rated, interview-based, or other independently obtained measures to reduce the influence of shared measurement methods.

Third, the study was conducted in a non-clinical community sample; therefore, the findings should be interpreted as preliminary psychometric evidence rather than as evidence of diagnostic utility or clinical screening performance. The study did not include structured psychiatric interviews, criterion validation procedures, or analyses of diagnostic sensitivity and specificity. Further validation in clinical populations is required before conclusions regarding diagnostic accuracy or screening performance can be drawn.

Fourth, the cross-sectional design did not permit assessment of the test–retest reliability or longitudinal stability of PHQ-9 scores over time.

Fifth, although confirmatory factor analysis was performed based on previously established theoretical models of the PHQ-9, complementary psychometric approaches, including item response theory and bifactor analyses, were not conducted. Item response theory could provide additional information regarding item-level performance across the underlying symptom continuum, while bifactor models could help determine the relative contribution of a general factor and specific symptom domains. Future studies should apply these approaches to further investigate the dimensionality and item-level properties of the Kazakh version of the PHQ-9.

Although expert review and cognitive debriefing provided preliminary evidence regarding item relevance, conceptual equivalence, and comprehensibility, comprehensiveness was not formally evaluated as a separate component of content validity. Accordingly, the evidence for content validity should be interpreted as preliminary.

## 5. Conclusions

This study provides preliminary psychometric evidence supporting the culturally adapted Kazakh version of the PHQ-9 as a reliable and structurally valid instrument for assessing depressive symptom severity in Kazakh-speaking community populations. By providing a standardized Kazakh-language measure, the study addresses an important gap in mental health assessment in Kazakhstan and contributes to international cross-cultural psychometric research. The findings support its use in community-based assessment and research, while its diagnostic accuracy and clinical screening utility have not yet been established. Future studies should evaluate criterion validity against structured clinical interviews, test–retest and longitudinal stability, responsiveness to change following therapeutic interventions, and measurement equivalence across demographic groups and different linguistic and cultural populations.

## Figures and Tables

**Table 1:** Item descriptive statistics for the PHQ-9 (*n* = 503).

Item	Mean	SD	Skewness	Kurtosis
PHQ1	0.476	0.737	1.574	2.031
PHQ2	0.478	0.728	1.540	1.955
PHQ3	0.566	0.822	1.383	1.127
PHQ4	0.712	0.845	1.083	0.511
PHQ5	0.402	0.736	1.957	3.349
PHQ6	0.378	0.748	2.043	3.420
PHQ7	0.444	0.756	1.818	2.835
PHQ8	0.274	0.596	2.486	6.529
PHQ9	0.112	0.400	4.099	18.510

**Table 2:** Internal consistency and corrected item–total correlations for the Kazakh version of the PHQ-9.

Item	Corrected Item–Total Correlation
PHQ1	0.68
PHQ2	0.75
PHQ3	0.69
PHQ4	0.75
PHQ5	0.69
PHQ6	0.76
PHQ7	0.69
PHQ8	0.70
PHQ9	0.45

Cronbach’s α  =  0.91. Corrected item–total correlations represent correlations between each item and the total scale score, excluding that item.

**Table 3:** Goodness-of-fit indices for the one- and two-factor confirmatory factor analysis models of the Kazakh version of the PHQ-9.

Model	χ^2^	df	CFI	TLI	RMSEA (90% CI)	SRMR
One-factor model	58.245	27	0.998	0.997	0.048 (0.031–0.065)	0.052
Two-factor model	53.251	26	0.998	0.998	0.046 (0.028–0.063)	0.050

CFI: Comparative Fit Index; TLI: Tucker–Lewis Index; RMSEA: Root-Mean-Square Error of Approximation; CI: confidence interval; SRMR: Standardized Root-Mean-Square Residual.

**Table 4:** Standardized factor loadings for the two-factor CFA model of the PHQ-9.

Item	Factor	Standardized Loading
PHQ1	Cognitive–affective	0.829
PHQ2	Cognitive–affective	0.890
PHQ6	Cognitive–affective	0.896
PHQ7	Cognitive–affective	0.838
PHQ8	Cognitive–affective	0.866
PHQ9	Cognitive–affective	0.752
PHQ3	Somatic	0.827
PHQ4	Somatic	0.904
PHQ5	Somatic	0.851

**Table 5:** Measurement invariance across genders for the two-factor PHQ-9 model.

Model	CFI	TLI	RMSEA	SRMR	ΔCFI	ΔRMSEA	ΔSRMR
Configural	0.997	0.996	0.059	0.055	-	-	-
Metric	0.996	0.995	0.067	0.070	−0.001	+0.008	+0.015
Scalar (equal loadings and thresholds)	0.998	0.998	0.046	0.055	+0.002	−0.021	−0.015

CFI: Comparative Fit Index; TLI: Tucker–Lewis Index; RMSEA: Root-Mean-Square Error of Approximation; SRMR: Standardized Root-Mean-Square Residual. Changes were calculated relative to the immediately preceding, less constrained model. Negative ΔCFI values and positive ΔRMSEA or ΔSRMR values indicate deterioration in model fit, whereas changes in the opposite direction indicate improvement.

**Table 6:** Comparison of psychometric properties of the PHQ-9 across validation studies.

Study	Country	Population	Sample Size	Cronbach’s α	Factor Structure
Kroenke et al. 2001 [3]	USA	Primary care patients	6000+	0.89	One factor
Wang et al. 2014 [12]	China	General population	553	0.86	Two factors
Richardson & Richards 2008 [13]	UK	Clinical sample	317	0.89	Two factors
Present study	Kazakhstan	General population	503	0.91	One- and two-factor models supported

## Data Availability

The original contributions presented in this study are included in the article/Supplementary Material. Further inquiries can be directed to the corresponding author.

## Supplementary Materials

The following supporting information can be downloaded at: https://www.mdpi.com/article/10.3390/healthcare14172713/s1. Table S1. Forward Translation of the PHQ-9 into Kazakh. Table S2. Reconciliation Table of the PHQ-9 Translation. Table S3. Back-Translation Comparison of the PHQ-9. Table S4. Cognitive Debriefing Results. File S1. Kazakh Version of the Patient Health Questionnaire-9 (PHQ-9).
