# Supplementary material for: Psychometric Validation of the Kazakh Version of the Patient Health Questionnaire-9 (PHQ-9)
Source: Healthcare (Basel). 2026 Aug 25;14(17):2713. doi: 10.3390/healthcare14172713 (PMC13565277; doi:10.3390/healthcare14172713)
Supplement: Supplementary file 1 [file healthcare-14-02713-s001.zip › healthcare-4456984-supplementary.pdf]

This supplementary material provides detailed information on the translation, cultural adaptation, and validation process of the Kazakh version of the Patient Health Questionnaire-9 (PHQ-9), including forward translation, reconciliation, back-translation, and cognitive debriefing results.

Supplementary Materials

Table S1. Forward Translation of the PHQ-9 into Kazakh

Item No.	Original English Version	T1 – Clinical Translator	T2 – Linguistic Translator	Translator Comments
Title	Patient Health Questionnaire-9	Науқастың денсаулық жағдайын бағалау сауалнамасы – 9 (PHQ-9)	НАУҚАСТЫҢ ДЕНСАУЛЫҚ ЖАҒДАЙЫН БАҒАЛАУ САУАЛНАМАСЫ-9 (PHQ-9)	
Instruction	Over the last two weeks, how often have you been bothered by any of the following problems?	Соңғы 2 апта ішінде төменде көрсетілген белгілердің қайсысы Сізде қаншалықты жиі байқалды?	Соңғы 2 апта ішінде төменде көрсетілген мәселелердің қайсысы Сізді қаншалықты жиі мазалады?	
Response 0	Not at all	Мүлдем болмады	Мүлдем мазаламады	
Response 1	Several days	Бірнеше күн	Бірнеше күн	
Response 2	More than half the days	Күндердің жартысынан көбі	Күндердің жартысынан көбі	
Response 3	Nearly every day	Күн сайын дерлік	Күн сайын	
Item 1	Little interest or pleasure in doing things	Күнделікті іс-әрекеттерге қызығушылықтың төмендеуі немесе олардан ләззат алмау	Іс-әрекеттерге қызығушылықтың немесе олардан ләззат алудың төмендеуі	
Item 2	Feeling down, depressed, or hopeless	Көңіл-күйдің төмендеуі, жабырқаңқылық, үмітсіздік сезімі	Көңіл-күйдің төмендеуі, жабырқаңқылық немесе үмітсіздік сезімі	
Item 3	Trouble falling or staying asleep, or sleeping too much	Ұйқы бұзылыстары: ұйқыға кетудің қиындауы, түнде жиі ояну немесе шамадан тыс ұйықтау	Ұйқыға кетудің қиындауы, ұйқыны ұстап тұра алмау немесе шамадан тыс ұйықтау	

Item 4	Feeling tired or having little energy	Айқын шаршағыштық, әлсіздік немесе энергияның төмендеуі	Шаршағыштық немесе энергияның жетіспеуі	
Item 5	Poor appetite or overeating	Тәбеттің төмендеуі немесе керісінше, шамадан тыс тамақтану	Тәбеттің төмендеуі немесе шамадан тыс тамақтану	
Item 6	Feeling bad about yourself — or that you are a failure or have let yourself or your family down	Өзіңізге қатысты жағымсыз баға беру, өзін сәтсіз адам ретінде сезіну немесе өзіңізді не отбасыңызды ақтамадым деп ойлау	Өзіңізді жаман сезіну, өзіңізді сәтсіз адам ретінде қабылдау немесе өзіңізді не отбасыңызды көңілден шығармадым деп ойлау	
Item 7	Trouble concentrating on things, such as reading the newspaper or watching television	Зейін шоғырландырудың қиындауы (мысалы, оқу, теледидар қарау кезінде)	Зейін қоюдың қиындауы (мысалы, газет оқу немесе теледидар көру кезінде)	
Item 8	Moving or speaking so slowly that other people could have noticed? Or the opposite — being so fidgety or restless that you have been moving around a lot more than usual	Психомоторлық тежелу (қимыл-қозғалыс пен сөйлеудің баяулауы, айналадағылар байқай алатындай) немесе психомоторлық қозу (әдеттегіден айқын мазасыздық, тынымсыздық)	Қозғалыс пен сөйлеудің баяулауы (мұны басқа адамдар байқауы мүмкін) немесе, керісінше, әдеттегіден әлдеқайда мазасыз, тынымсыз болу	
Item 9	Thoughts that you would be better off dead, or of hurting yourself in some way	Өмір сүрудің мәні жоқ деген ойлар, өлгенім дұрыс болар еді деген ойлар немесе өз-өзіңізге зиян келтіру туралы ойлар	Өлгенім жақсы болар еді деген ойлар немесе өз-өзіңізге қандай да бір түрде зиян келтіру туралы ойлар	
Additional	If you checked off any problems, how difficult have these problems made it for you to do your work, take care of things at home, or get along with other people?	Егер жоғарыда аталған белгілердің кемінде біреуі байқалса, олар Сіздің кәсіби қызметіңізді атқаруыңызға, тұрмыстық міндеттерді орындауыңызға немесе басқа адамдармен қарым-қатынас орнатуыңызға қаншалықты дәрежеде қиындық келтірді?	Егер Сіз қандай да бір мәселелерді белгілеген болсаңыз, бұл мәселелер жұмысыңызды орындауыңызға, үйдегі істермен айналысуыңызға немесе басқа адамдармен қарым-қатынас жасауыңызға қаншалықты қиындық туғызды?	

		R. L. Spitzer, J. B. W. Williams, K. Kroenke және әріптестері.	Dr. Robert L. Spitzer, Janet B. W. Williams, Kurt Kroenke және әріптестері.	
		Аудару және таратуға шектеу қойылмайды.	Pfizer Inc. компаниясының білім беру грантының қолдауымен әзірленген.	
			Көбейтуге, аударуға, көрсетуге және таратуға рұқсат талап етілмейді.	

Table S2. Reconciliation Table of the PHQ-9 Translation

Item No.	Original English Version	T1 – Clinical Translator	T2 – Linguistic Translator	Final Reconciled Version (Kazakh)	Decision Rationale / Notes
Title	Patient Health Questionnaire-9	Науқастың денсаулық жағдайын бағалау сауалнамасы – 9 (PHQ-9)	НАУҚАСТЫҢ ДЕНСАУЛЫҚ ЖАҒДАЙЫН БАҒАЛАУ САУАЛНАМАСЫ-9 (PHQ-9)	Науқастың денсаулық жағдайын бағалау сауалнамасы – 9 (PHQ-9)	Final wording selected to preserve original meaning and ensure clarity and comprehensibility for the general population
Instruction	Over the last two weeks, how often have you been bothered by any of the following problems?	Соңғы 2 апта ішінде төменде көрсетілген белгілердің қайсысы Сізде қаншалықты жиі байқалды?	Соңғы 2 апта ішінде төменде көрсетілген мәселелердің қайсысы Сізді қаншалықты жиі мазалады?	Соңғы екі апта ішінде төменде көрсетілген мәселелер Сізді қаншалықты жиі мазалады?	Final wording selected to preserve original meaning and ensure clarity and comprehensibility for the general population

Response 0	Not at all	Мүлдем болмады	Мүлдем мазаламады	Мүлде болмады	Response options were selected to reflect the original frequency gradation and ensure consistent interpretation across items
Response 1	Several days	Бірнеше күн	Бірнеше күн	Бірнеше күн	Response options were selected to reflect the original frequency gradation and ensure consistent interpretation across items
Response 2	More than half the days	Күндердің жартысынан көбі	Күндердің жартысынан көбі	Күндердің көбінде	Response options were selected to reflect the original frequency gradation and ensure consistent

					interpretation across items
Response 3	Nearly every day	Күн сайын дерлік	Күн сайын	Күн сайын дерлік	Response options were selected to reflect the original frequency gradation and ensure consistent interpretation across items
Item 1	Little interest or pleasure in doing things	Күнделікті іс-әрекеттерге қызығушылықтың төмендеуі немесе олардан ләззат алмау	Іс-әрекеттерге қызығушылықтың немесе олардан ләззат алудың төмендеуі	Істерге қызығушылықтың немесе олардан ләззат алудың төмендеуі	Final wording combines clinical accuracy and linguistic clarity, prioritizing conceptual equivalence over literal translation

Item 2	Feeling down, depressed, or hopeless	Көңіл-күйдің төмендеуі, жабырқаңқылық, үмітсіздік сезімі	Көңіл-күйдің төмендеуі, жабырқаңқылық немесе үмітсіздік сезімі	Өзіңізді жабырқаңқы, көңілсіз немесе үмітсіз сезіну	Final wording combines clinical accuracy and linguistic clarity, prioritizing conceptual equivalence over literal translation
Item 3	Trouble falling or staying asleep, or sleeping too much	Ұйқы бұзылыстары: ұйқыға кетудің қиындауы, түнде жиі ояну немесе шамадан тыс ұйықтау	Ұйқыға кетудің қиындауы, ұйқыны ұстап тұра алмау немесе шамадан тыс ұйықтау	Ұйықтауға қиналу, ұйқының жиі бұзылуы немесе шамадан тыс ұйықтау	Final wording combines clinical accuracy and linguistic clarity, prioritizing conceptual equivalence over literal translation
Item 4	Feeling tired or having little energy	Айқын шаршағыштық, әлсіздік немесе энергияның төмендеуі	Шаршағыштық немесе энергияның жетіспеуі	Шаршау немесе күш-қуаттың азаюы	Final wording combines clinical accuracy and linguistic clarity, prioritizing conceptual equivalence

					over literal translation
Item 5	Poor appetite or overeating	Тәбеттің төмендеуі немесе керісінше, шамадан тыс тамақтану	Тәбеттің төмендеуі немесе шамадан тыс тамақтану	Тәбеттің төмендеуі немесе шамадан тыс тамақтану	Final wording combines clinical accuracy and linguistic clarity, prioritizing conceptual equivalence over literal translation
Item 6	Feeling bad about yourself — or that you are a failure or have let yourself or your family down	Өзіңізге қатысты жағымсыз баға беру, өзін сәтсіз адам ретінде сезіну немесе өзіңізді не отбасыңызды ақтамадым деп ойлау	Өзіңізді жаман сезіну, өзіңізді сәтсіз адам ретінде қабылдау немесе өзіңізді не отбасыңызды көңілден шығармадым деп ойлау	Өзіңізді сәтсіз адам ретінде сезіну немесе өзіңізді не отбасыңызды жерге қараттым деп ойлау	Neutral wording was selected to avoid stigmatization and moral judgment while preserving the self-evaluation component of the original item

Item 7	Trouble concentrating on things, such as reading the newspaper or watching television	Зейін шоғырландырудың қиындауы (мысалы, оқу, теледидар қарау кезінде)	Зейін қоюдың қиындауы (мысалы, газет оқу немесе теледидар көру кезінде)	Зейінді шоғырландырудың қиындауы (мысалы, газет оқу немесе теледидар көру кезінде)	Examples were retained to facilitate comprehension, consistent with the original PHQ-9 structure
Item 8	Moving or speaking so slowly that other people could have noticed? Or the opposite — being so fidgety or restless that you have been moving around a lot more than usual	Психомоторлық тежелу (қимыл-қозғалыс пен сөйлеудің баяулауы, айналадағылар байқай алатындай) немесе психомоторлық қозу (әдеттегіден айқын мазасыздық, тынымсыздық)	Қозғалыс пен сөйлеудің баяулауы (мұны басқа адамдар байқауы мүмкін) немесе, керісінше, әдеттегіден әлдеқайда мазасыз, тынымсыз болу	Қимыл-қозғалысыңыздың немесе сөйлеуіңіздің баяулауы (оны басқа адамдар байқауы мүмкін) немесе, керісінше, әдеттегіден әлдеқайда мазасыз, тынымсыз болуыңыз	Final wording preserves the bipolar structure of psychomotor retardation and agitation while ensuring linguistic naturalness
Item 9	Thoughts that you would be better off dead, or of hurting yourself in some way	Өмір сүрудің мәні жоқ деген ойлар, өлгенім дұрыс болар еді деген ойлар немесе өз-өзіңізге зиян келтіру туралы ойлар	Өлгенім жақсы болар еді деген ойлар немесе өз-өзіңізге қандай да бір түрде зиян келтіру туралы ойлар	Өлгенім жақсы болар еді деген ойлардың пайда болуы немесе өзіңізге қандай да бір түрде зиян келтіру туралы ойлар	Careful neutral wording was selected to reflect the original meaning without intensifying or minimizing suicidal content, in line with international

					screening standards
Additional	If you checked off any problems, how difficult have these problems made it for you to do your work, take care of things at home, or get along with other people?	Егер жоғарыда аталған белгілердің кемінде біреуі байқалса, олар Сіздің кәсіби қызметіңізді атқаруыңызға, тұрмыстық міндеттерді орындауыңызға немесе басқа адамдармен қарым-қатынас орнатуыңызға қаншалықты дәрежеде қиындық келтірді?	Егер Сіз қандай да бір мәселелерді белгілеген болсаңыз, бұл мәселелер жұмысыңызды орындауыңызға, үйдегі істермен айналысуыңызға немесе басқа адамдармен қарым-қатынас жасауыңызға қаншалықты қиындық туғызды?	Егер жоғарыда көрсетілген мәселелердің кемінде біреуі байқалса, олар Сіздің жұмысыңызды атқаруыңызға, үйдегі істерді орындауыңызға немесе басқа адамдармен қарым-қатынас жасауыңызға қаншалықты қиындық туғызды?	Wording was selected to ensure functional relevance across occupational and non-occupational contexts

Table S3. Back-Translation Comparison of the PHQ-9

Item No.	Original English Version	Back-translation 1	Back-translation 2	Discrepancy Type (A/B/C)	Comments / Action Taken
Title	Patient Health Questionnaire-9	Patient Health Questionnaire – 9 (PHQ-9)	Patient Health Questionnaire-9 (PHQ-9)	A	No conceptual discrepancy; wording differences acceptable.

Instruction	Over the last two weeks, how often have you been bothered by any of the following problems?	Over the past two weeks, how often have you been bothered by the following problems?	During the last two weeks, how often have you been bothered by any of the following problems?	A	No conceptual discrepancy; wording differences acceptable.
Response 0	Not at all	Not at all	Not at all	A	No conceptual discrepancy; wording differences acceptable.
Response 1	Several days	Several days	Several days	A	No conceptual discrepancy; wording differences acceptable.
Response 2	More than half the days	More than half the days	More than half the days	A	No conceptual discrepancy; wording differences acceptable.
Response 3	Nearly every day	Nearly every day	Nearly every day	A	No conceptual discrepancy; wording differences acceptable.
Item 1	Little interest or pleasure in doing things	Diminished interest or pleasure in activities	Little interest or pleasure in doing things	A	No conceptual discrepancy; wording differences acceptable.

Item 2	Feeling down, depressed, or hopeless	Feeling down, depressed, or hopeless	Feeling sad, low, or hopeless	A	No conceptual discrepancy; wording differences acceptable.
Item 3	Trouble falling or staying asleep, or sleeping too much	Difficulty falling asleep, frequent sleep disturbances, or excessive sleeping	Trouble falling asleep, staying asleep, or sleeping too much	B	Minor wording variation; conceptual meaning preserved. No change required.
Item 4	Feeling tired or having little energy	Fatigue or decreased energy	Feeling tired or having little energy	A	No conceptual discrepancy; wording differences acceptable.
Item 5	Poor appetite or overeating	Poor appetite or overeating	Poor appetite or eating too much	A	No conceptual discrepancy; wording differences acceptable.
Item 6	Feeling bad about yourself — or that you are a failure or have let yourself or your family down	Feeling like a failure or believing you have let yourself or your family down	Feeling bad about yourself — or that you are a failure or have let yourself or your family down	A	No conceptual discrepancy; wording differences acceptable.
Item 7	Trouble concentrating on things, such as reading the newspaper or watching television	Difficulty concentrating (for example, while reading a newspaper or watching television)	Trouble concentrating on things, such as reading the newspaper or watching TV	A	No conceptual discrepancy; wording differences acceptable.

Item 8	Moving or speaking so slowly that other people could have noticed? Or the opposite — being so fidgety or restless that you have been moving around a lot more than usual	Noticeable slowing of movements or speech (observable by others), or, conversely, increased restlessness and agitation compared to usual	Moving or speaking so slowly that other people could notice, or the opposite — being unusually fidgety or restless	A	No conceptual discrepancy; wording differences acceptable.
Item 9	Thoughts that you would be better off dead, or of hurting yourself in some way	Thoughts that it would be better to be dead or thoughts of harming yourself in some way	Thoughts that you would be better off dead or of hurting yourself in some way	A	No conceptual discrepancy; wording differences acceptable.
Functional item	If you checked off any problems, how difficult have these problems made it for you to do your work, take care of things at home, or get along with other people?	If you marked at least one problem above, how difficult have these problems made it for you to perform your work, manage household responsibilities, or interact with other people?	If you checked any problems, how difficult have they made it for you to do your work, take care of things at home, or get along with other people?	A	No conceptual discrepancy; wording differences acceptable.

Table S4. Cognitive Debriefing Results

Item	Main feedback themes	Number of participants reporting issues	Type of issue (L/C/CU)	Decision	Final action
PHQ-1	Generally well understood	0	—	No change	Retained as final version
PHQ-2	Generally well understood	0	—	No change	Retained as final version

PHQ-3	Generally well understood	0	—	No change	Retained as final version
PHQ-4	Generally well understood	0	—	No change	Retained as final version
PHQ-5	Generally well understood	0	—	No change	Retained as final version
PHQ-6	Generally well understood	0	—	No change	Retained as final version
PHQ-7	Generally well understood	0	—	No change	Retained as final version
PHQ-8	Generally well understood	0	—	No change	Retained as final version
PHQ-9	Generally well understood	0	—	No change	Retained as final version
GAD-1	Generally well understood	0	—	No change	Retained as final version
GAD-2	Generally well understood	0	—	No change	Retained as final version
GAD-3	Generally well understood	0	—	No change	Retained as final version
GAD-4	Generally well understood	0	—	No change	Retained as final version
GAD-5	Generally well understood	0	—	No change	Retained as final version
GAD-6	Generally well understood	0	—	No change	Retained as final version
GAD-7	Generally well understood	0	—	No change	Retained as final version
Response options	Generally well understood	0	—	No change	Retained as final version

Instructions	Generally well understood	0	—	No change	Retained as final version

File S1. Kazakh Version of the Patient Health Questionnaire-9 (PHQ-9)

Соңғы екі апта ішінде төменде көрсетілген мәселелер Сізді қаншалықты жиі мазалады?

№	Сұрақтар
1	Істерге қызығушылықтың немесе олардан ләззат алудың төмендеуі

2	Өзіңізді жабырқаңқы, көңілсіз немесе үмітсіз сезіну
3	Ұйықтауға қиналу, ұйқының жиі бұзылуы немесе шамадан тыс ұйықтау
4	Шаршау немесе күш-қуаттың азаюы
5	Тәбеттің төмендеуі немесе шамадан тыс тамақтану
6	Өзіңізді сәтсіз адам ретінде сезіну немесе өзіңізді не отбасыңызды жерге қараттым деп ойлау
7	Зейінді шоғырландырудың қиындауы (мысалы, газет оқу немесе теледидар көру кезінде)
8	Қимыл-қозғалысыңыздың немесе сөйлеуіңіздің баяулауы (оны басқа адамдар байқауы мүмкін) немесе, керісінше, әдеттегіден әлдеқайда мазасыз, тынымсыз болуыңыз
9	Өлгенім жақсы болар еді деген ойлардың пайда болуы немесе өзіңізге қандай да бір түрде зиян келтіру туралы ойлар

Жауап нұсқалары

**Ұпай**	**Жауап**
0	Мүлде болмады

1	Бірнеше күн
2	Күндердің көбінде
3	Күн сайын дерлік

Қосымша сұрақ
Егер жоғарыда көрсетілген мәселелердің кемінде біреуі байқалса, олар Сіздің жұмысыңызды атқаруыңызға, үйдегі істерді орындауыңызға немесе басқа адамдармен қарым-қатынас жасауыңызға қаншалықты қиындық туғызды?

**Жауап**
Мүлде қиын емес
Біраз қиын
Өте қиын
Аса қиын

Note
The PHQ-9 was originally developed by Kroenke, Spitzer, and Williams (2001) and is widely used for screening depressive symptoms in clinical and research settings. The Kazakh version presented here was translated and culturally adapted following internationally recommended cross-cultural adaptation procedures.
